# Follow-Up of Elevated Blood Lead Levels and Sources in a Cohort of Children in Benin

**DOI:** 10.3390/ijerph17228689

**Published:** 2020-11-23

**Authors:** Shukrullah Ahmadi, Barbara Le Bot, Roméo Zoumenou, Séverine Durand, Nadine Fiévet, Pierre Ayotte, Achille Massougbodji, Maroufou Jules Alao, Michel Cot, Philippe Glorennec, Florence Bodeau-Livinec

**Affiliations:** 1 Centre of Research in Epidemiology and Statistics (CRESS), Institut National de la Sante et de la Recherche Medicale (INSERM), Institut National de la Recherche Agronomique (INRA), Université de Paris, F-75004 Paris, France; 2 Ecole des Hautes Études en Santé Publique (EHESP), Institut National de la Sante et de la Recherche Medicale (Inserm), Institut de Recherche en Santé, Environnement et Travail (Irset)—UMR_S 1085, University of Rennes, F-35000 Rennes, France; barbara.lebot@ehesp.fr (B.L.B.); severine.durand@ehesp.fr (S.D.); philippe.glorennec@ehesp.fr (P.G.); 3 Institut de Recherche pour le Développement (IRD), Paris Descartes Université, 75006 Paris, France; zoumenour@yahoo.fr (R.Z.); nadine.fievet@ird.fr (N.F.); michel.cot@ird.fr (M.C.); 4 Axe Santé des Populations et Pratiques Optimales en Santé, Centre de Recherche du CHU de Québec, Université Laval—Institut National de Santé Publique du Québec, Quebec City, QC G1V 5B3, Canada; pierre.ayotte@inspq.qc.ca; 5 Faculté des Sciences de la Santé, 01 BP 526 Cotonou, Benin; massougbodjiachille@yahoo.fr; 6 Paediatric Service, Mother and Child Teaching Hospital (CHU-MEL), 01 BP 107 Cotonou, Benin; amomj@yahoo.fr; 7 Ecole des Hautes Études en Santé Publique (EHESP), Obstetrical, Perinatal and Pediatric Epidemiology Research Team (EPOPé)—UMR1153, F-35000 Rennes, France; florence.bodeau-livinec@ehesp.fr

**Keywords:** lead poisoning, environmental health, toxic metals, Sub-Saharan Africa, environmental exposure

## Abstract

Lead exposure is associated with poor cognitive development in children. Very few studies in sub-Saharan Africa (SSA) have studied blood lead levels (BLLs) and non-gasoline sources of exposure in children. Data from a birth cohort in Benin (2011–2013) suggested that 58% of 1-year-old children had BLLs > 50 ug/L. We aimed to investigate the prevalence of elevated BLLs (>50 µg/L and >100 µg /L) among 425 of these children at 6 years of age in 2016–2018 and to compare BLLs between age 1 and 6 years, and study sources of lead at age 6 years. BLLs were analysed by inductively coupled plasma mass spectrometry. Multiple linear regression and quantile regressions were used to study potential sources of lead. The prevalence of BLLs > 50 µg/L in children was 59.5% (Geometric Mean (GM) 56.4 µg/L, 95% CI: 54.1–58.7) at 6 years of age compared to 54.8% (GM 56.5 µg/L, 95% CI: 53.4–59.6) at 1 year of age. The prevalence of children with BLLs > 100 µg/L decreased from 14.4% at 1 year of age to 8.2% at 6 years of age. After adjustment for all other covariates, consumption of peanuts more than once per month was significantly associated with a 22.0% (95% CI: 4.6, 42.5) increment in BLLs at age 6 years compared with no consumption. Consumption of bushmeat killed by lead bullets at age 6 years was associated with an increase in the higher percentiles of BLLs (P75) compared with the absence of this source. Other potential sources of lead associated with BLLs with marginal significance were consumption of rice, paternal occupational exposure, and the presence of activity with the potential use of lead. This prospective cohort confirms the persistently high prevalence of elevated BLLs in children residing in a rural region in the south of Benin, as well as the presence of multiple and continuous sources of lead. These results highlight the need for prevention programs to reduce and eliminate lead exposure in children.

## 1. Introduction

Lead exposure causes a wide range of adverse health effects in children, including poor cognitive development. Lead exposure in early childhood is also associated with later adverse health outcomes in later childhood, adolescence, and adult age. Higher blood lead levels (BLLs) and higher lead concentrations in bones and teeth have been associated with behavioural problems, poor educational outcomes, and reduced adult brain volume [1,2]. Furthermore, lead, a cumulative pollutant, may have consequences throughout one’s life course, from infancy to adulthood [3]. In 2019, lead exposure accounted for 0.9 million deaths and 21.7 million years of disability-adjusted life years (DALYs) worldwide due to long-term effects on health [4]. Low-income countries suffered the highest burden.

The U.S. Centers for Disease Control and Prevention (CDC) has set blood lead reference value (BLRV) of 50 μg/L—corresponding to the 97.5th percentile of the blood lead levels (BLLs) distribution among children 1–5 years old in the United States (U.S.) [5,6]. However, there is no known safe lead exposure in children as there is mounting evidence suggesting that lower BLLs, i.e., BLLs lower than 50 μg/L may be associated with a reduced cognitive capacity [1,7,8]. Based on the benchmark dose (BMD) estimates of the European Food Safety Authority (EFSA), BLLs of 12 µg/L may lead to a loss of one intelligence quotient (IQ) point in children [9].

BLLs in children have decreased considerably over the last three to four decades in high-income nations, particularly in the U.S. and in Europe [10,11,12,13]. This has been achieved through historical regulations, such as bans on leaded gasoline, together with other control measures, such as controlling the use of lead in commercial products, such as paint [10,11,12]. Leaded gasoline was officially phased out in most sub-Saharan African (SSA) countries by the end of 2005 [14]. This resulted in significant reductions in BLLs in some African countries in the past one-and-half decades, especially in South Africa and Uganda [15,16], but not in others [16,17]. Concerted efforts have been made through inter-governmental conventions to eliminate the use of lead in paint, as per the objective of the Global Alliance to Eliminate Lead Paint (GAELP) [18], aimed to globally eliminate lead in paint by 2020. However, lead-based paints are still considered a serious threat to public health due to their continuing wide use in many low- and middle-income countries [19,20]. As of 2019, Benin, like some neighbouring West African countries, may have not fully implemented laws to control lead in paint [18]. Therefore, lead exposure continues to be a public health problem in developing countries due to the inadequacy of regulations or the inability to enforce regulations to control lead exposure [21,22,23].

An understanding of the sources of exposure is crucial to the development and implementation of regulations to control and reduce exposures [10]. However, very few studies have studied BLLs and sources of exposure in children after the phase-out of leaded gasoline in SSA [15,16,24,25,26]. Data from a birth cohort in Benin (2011–2013) showed that 58% of 1-year-old children had BLLs >50 ug/L [26]. In addition to the presence of paint in the house, one of the potential sources of lead was the consumption of bushmeat hunted by lead bullets [27], which was found to be associated with higher BLLs [26,28]. Hunting, sale and consumption of bushmeat were banned in West Africa following the 2013–2016 epidemic of Ebola Virus Disease (EVD) [29]. However, change in bushmeat consumption and subsequent association with elevated BLLs in children after these regulations are unclear.

We aimed to investigate BLLs in the same children at 6 years of age in 2016–2018 in a post-EVD context, after the 2014–2016 ban of bushmeat consumption and official phase-out (2005) of leaded gasoline. Precisely, this study aimed to evaluate the prevalence of elevated BLLs i.e., BLLs beyond 50 µg/L, and beyond 100 µg /L among children in a semi-rural setting in Benin and to identify sources of lead exposure. We also aimed to compare the prevalence of elevated BLLs and potential sources of lead in children at age 1 and 6 years.

## 2. Materials and Methods

### 2.1. Study Design and Population

**Figure 1:**
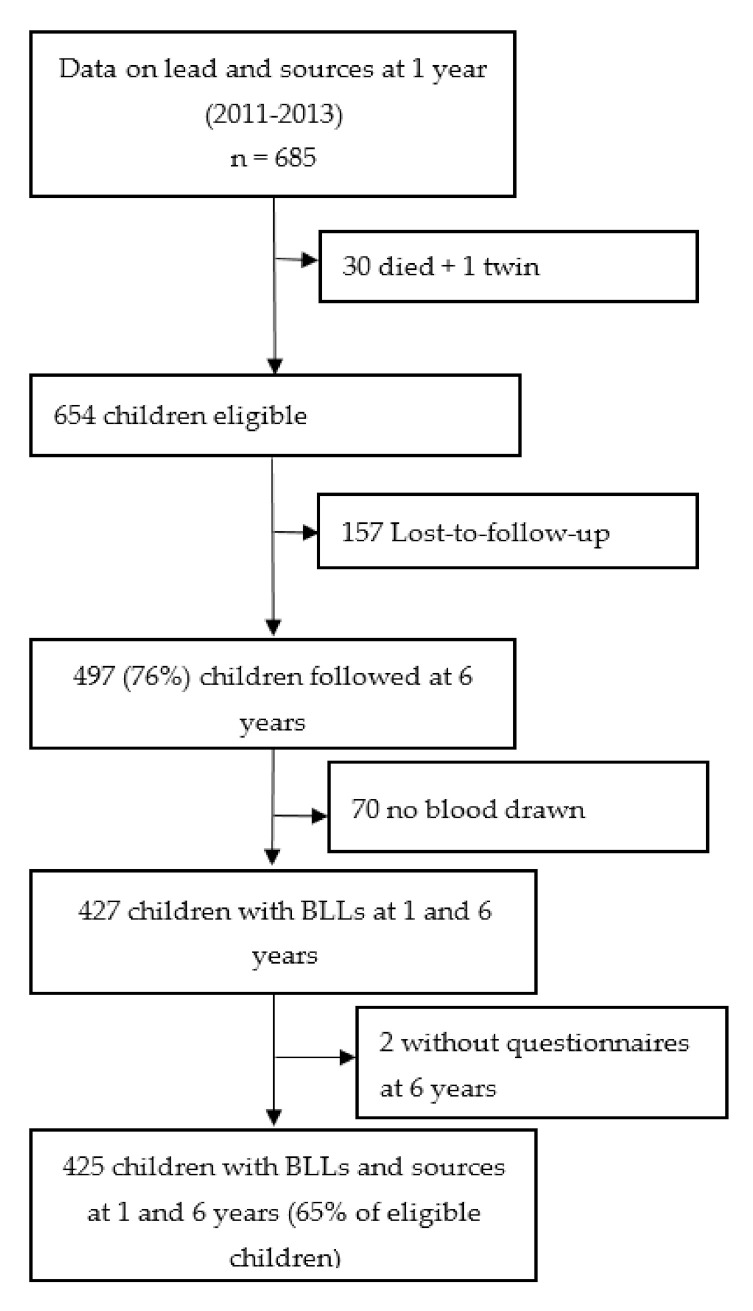
Summary of participant selection. BLLs = blood lead levels.

The study included children who were born to pregnant women enrolled in the “Malaria in Pregnancy Preventive Alternative Drugs” (MiPPAD) clinical trial (NCT00811421), comparing two intermittent preventive treatments of malaria in pregnancy [30]. These children were followed at 1 and 6 years of age. At 1 year of age, children (*n* = 685) were investigated for BLLs, potential sources of lead, and psychomotor development in the TOVI study (in Fon language *Tovi* means child from the country) [26]. At 6 years of age, they were followed in the EXPLORE study (2016–2018) to reassess BLLs, potential sources, and neurocognitive development. The study took place in three health centres (Allada, Attogon, and Sekou) in the district of Allada, a semi-rural district located in the South of Benin. In total, 425 children with data on both BLLs and sources of lead at 1 and 6 years of age were included in the analyses (Figure 1).

### 2.2. Exposure and Relevant Data Collection

Information on data collection at 1 year of age is described in detail elsewhere [26]. Potential sources of lead were investigated by administering a structured questionnaire. The potential sources of lead investigated included, but not limited to, were related to parents, family/housing, and child characteristics. Paternal and maternal characteristics included paternal occupation (parental risk of occupational exposure was developed from the type of paternal occupation [31]); activities in the house or neighbourhood (activities included metal smelter, battery recycling/storage, radiator repair, metal recycling/storage, lead solder, vehicle repair, and manufacturing of ammunition or metal fish baits or metal objects); and maternal use of eye cosmetics (Khol). For Khol eye makeup, two (slightly different) questions were included in the questionnaire. At 1 year, the question included was “Do you sometimes wear makeup of Khol?” While at 6 years the question included was, “Do you sometimes wear makeup of Khol (black makeup)? Housing characteristics consisted of the presence of paint and paint chips in the house; house made up of mud; residence at 200 m of heavy traffic; type of cooking utensils (use of artisanal cookware made from recycled material and terracotta/clay); and source of drinking water (piped water, well water). Information on family wealth was collected through a checklist of material possession (such as a car, motorbike, bike, television, cow, and radio), which was later transformed into a wealth scale with scores ranging from 1 to 15 [26]. Child characteristics included, but were not limited to, consumption of several food types, including meat killed by lead ammunition; consumption of vegetables and tubers (peanuts, beans, rice and yam/sweet potatoes, manioc); and certain child behaviours (use of local eye cosmetics Khol, hand-to-mouth-behaviour, and ingestion of soil or other products, i.e., earth, kaolin, kalaba, termite mound). Two questions regarding bushmeat consumption were included in the questionnaire. First, the same question at 1 and 6 years was asked: “Does your child eat meat from animals killed by a rifle?” Besides, at 6 years of age, a question regarding current consumption was added: “Currently, does your child eat meat or poultry from animals killed by a rifle in a regular week?”.

### 2.3. Blood Sampling and Analysis

BLLs were analysed at the *Centre de Toxicologie, Institut National de Santé Publique du Québec* (INSPQ, Quebec City, QC, Canada) and Ecole des Hautes Études en Santé Publique (EHESP) laboratory in Rennes, France, at 1 year of age and 6 years of age, respectively. All BLLs were analysed by inductively coupled plasma mass spectrometry (ICP-MS) after dilution of blood samples, with, respectively, a detection limit at 0.2 µg/L and 2 µg/L. All results were higher than 2 µg/L. The analytical methods are described elsewhere [26,32].

### 2.4. Statistical Analysis

We described BLLs in children in terms of geometric mean, median, range, and prevalence of BLLs >50 µg/L and >100 µg/L. BLLs between boys and girls were compared using the Wilcoxon rank-sum test. The Wilcoxon rank-sum test was used to compare median BLLs between boys and girls. Prevalence of BLLs >50 µg/L and >100 µg/L between 1 and 6 years were compared using McNemar’s chi-square test.

To identify potential sources of exposure, we used ordinary least squares (OLS), i.e., linear regression and logistic regression in the bivariate analysis (Supplementary Table S2). For linear regression, BLLs was log-transformed to ensure normal distribution. The results of the linear regression (coefficients and confidence intervals) were presented in percent change of BLLs. A logistic regression using a cut-off of BLLs above the 90th percentile (93.3 µg/L) was used to identify potential sources of lead in the bivariate analyses. This cut-off was used in an attempt to not miss potential sources associated with higher exposure levels. Potential sources of exposure associated with BLLs with a *p*-value < 0.2 in the bivariate analyses either in the linear regression or logistic regression was included and controlled for in the multivariable models. These include consumption of meat harvested with lead bullets, consumption of rice, consumption of peanuts, consumption of sweet potatoes, presence of activity with the potential use of lead, presence of high or moderate risk of paternal occupational exposure, presence of paint in the house, and use of borehole/cement/dug well for the water source. Multivariable quantile regressions, including sources of exposure identified in bivariate analyses, were conducted at 25th, 50th, 75th, and 90th percentiles of BLLs using quantile regression (qreg) command in STATA [33]. Models were further adjusted for child sex and family wealth, as they were associated with BLLs at *p* < 0.20. Expected increases in BLLs are presented as an increment in BLLs at different percentiles of BLLs in the quantile regressions and as a percentage increase in BLLs in the linear regression, with 95% confidence intervals. All analyses were carried out on Stata version 14 [34]. The level of significance was set at *p* < 0.05.

A sensitivity analysis was also conducted by including all children (*n* = 478) with BLLs assessed at age 6 independently of the availability of data on BLLs and sources at age 1 year. This analysis consisted of both multivariable linear regression and quantile regression to examine associations between potential sources and BLLs and were adjusted for child sex and family wealth quartiles. The same potential sources, as described above in the main multivariable analysis, were included in these models.

## 3. Results

### 3.1. Population

The characteristics of the population are described in Table 1. The level of education was low, with 28.8% of fathers and 11.2% of mothers who had completed secondary education. The mean age of the children evaluated in 2016–2018 was 6.2 years. Most of the children (66.6%) attended school at 6 years of age. Only 4% of the parents were smokers.

### 3.2. Comparison between Children Included in the Analyses and Children Excluded

Characteristics of children included and excluded from the analyses are presented in the Supplementary Table S1. Comparison between children followed at 6 years of age and included in this analysis (*n* = 425) and children not included (*n* = 260) are shown in the Supplementary Table S1. Children included had higher socioeconomic status as compared to children excluded. Children excluded were more likely to present BLLs > 50 µg/L (62.7%) than children included (55.1%), but at the limit of statistical significance (*p* = 0.05). Besides, there was a lower proportion of children excluded who ate meat killed by lead ammunition (34.8%) compared with those followed (41.2%), but this was not statistically significant (*p* = 0.1).

### 3.3. Comparison of BLLs at Age 1 and 6 Years

**Figure 2:**
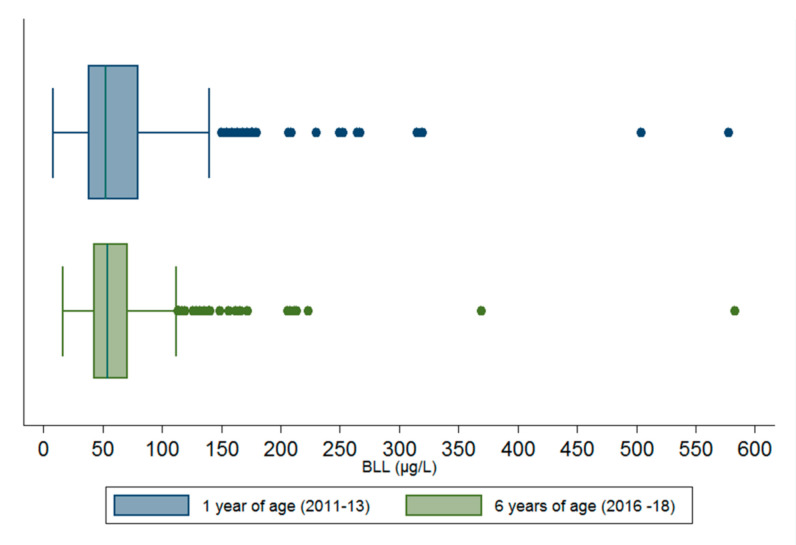
Comparison of blood lead level distribution in 2011–2013 and 2016–2018 (*n* = 425).

The geometric mean BLLs was 56.5 µg/L (95% CI: 53.4, 59.6) and 56.4 µg/L (95% CI: 54.1, 58.7 at 1 and 6 years of age, respectively. BLLs at age 1 and 6 years were correlated (r = 0.34, *p* < 0.001). The overall distribution of BLLs at 6 years (µg/L) in terms of percentiles were as follows: 34.7 µg/L (10th), 42.6 µg/L (25th), 54.2 µg/L (50th), 71 µg/L (75th), 93.3 µg/L (90th), 116.8 µg/L (95th). The proportion of children with BLLs > 50 µg/L at 6 years of age was found to be comparable to the prevalence of 54.8% at age 1 year (*p* = 0.12). However, the proportion of children with BLLs > 100 µg/L decreased between 1 and 6 years (14.4% at age 1 year vs. 8.2% at age 6 years, *p* < 0.01) (Figure 2).

### 3.4. Comparison of Potential Sources of Lead at Two Age Points

The potential source of exposure was frequent at the age of 6. There was no difference in terms of the frequencies of potential sources of lead exposure identified at one year of age (bushmeat consumption, drinking piped water, presence of paint in the house, presence of paint chips) between 1 and 6 years of age (Table 2), but for drinking piped water and maternal use of eye cosmetics, which increased at 6 years of age.

### 3.5. Associations between Sources of Lead and BLLs at Age 6 Years

In the bivariate analysis (Supplementary Table S2), the current consumption of bushmeat killed by lead bullets, consumption of peanuts more than once per month, eating rice more than four times per week, presence of activity involving potential use of lead in the household or the neighbourhood, and high or moderate parental risk of occupational exposure were associated with increased BLLs with *p*-value < 0.20 and then were included in the multivariable model. Consumption of sweet potatoes less than once per month was associated with decreased BLLs compared with no consumption.

Multivariable linear and quantile regression analysis (Table 3) further supported associations between potential sources of lead and BLLs. After adjustment for all other covariates, rice consumption was associated with BLLs: although not significant, the increase seemed to be higher for P50 and P75 of BLLs.

The current consumption of bushmeat killed by lead bullets was significantly associated with 13.3% (95% CI: 1.7, 26.2) increment in BLLs compared with no consumption of this source. The increment was higher for upper quartiles, although not always significant, adjusting for all other covariates in the model. Consumption of peanuts more than once per month was significantly associated with 22% (95% CI: 4.6, 42.5) increment in BLLs compared to never consumers. It contributed notably to a high increment, i.e., 73.0 (95% CI: 26.9; 119.1) µg/L on the 90th percentiles of BLLs as compared to never consumption. Overall, this means that both the consumption of bushmeat killed by ammunition and consumption of peanuts particularly contributed to high BLLs in children. Activity with the potential use of lead was associated with increased BLL at the limit of significance, especially for the higher quartile, although not significant.

These potential sources of lead were common in this semi-rural area: 9.8%, 66.7%, 18.8% of children reported eating peanuts more than once per month, rice more than four times per week, and meat, respectively.

The sensitivity analysis (Supplementary Table S4) on all children (*n* = 478), including those without data on BLLs, and sources at age 1 year confirmed these associations. In the multivariable linear regression, in addition to peanuts and bushmeat consumption, presence of activity with the potential use of lead in the household or neighbourhood was also significantly associated with 13.3% increment in BLLs (95% CI: 1.3; 26.6) as compared to the absence of this type of activity, adjusting for all other covariates in the model. Consumption of sweet potatoes was significantly associated with decreased BLLs on the 75th percentile of BLLs, whereas the significance of the association between the consumption of rice and BLLs was marginal.

### 3.6. Associations between Sources of Lead and Sociodemographic Factors

A further investigation between potential sources of lead and socio-demographic factors showed that only presence of paint in the house was associated with the highest family wealth score and maternal education (*p* < 0.05) (Supplementary Table S3), considering that presence of paint was associated with elevated BLLs in children at 1 year of age [26]. Consumption of bushmeat, consumption of rice, consumption of peanuts, presence of risk of occupational exposure and presence of activities involving potential use of lead were not associated with socio-demographic factors.

## 4. Discussion

The prevalence of BLLs > 50 µg/L in children was found to be comparable to the 1-year prevalence. However, the prevalence of children with BLLs beyond 100 µg/L significantly decreased at age 6 year assessed in 2016–2018 as compared to age 1 year assessed in 2011–2013. We confirmed that the consumption of bushmeat killed by lead bullets was associated with an increase in BLLs, as initially identified at 1 year of age [26]. Furthermore, we identified new sources including the consumption of peanuts.

This study extends the previous limited studies on lead exposure in children in SSA. In line with our findings, previous studies have shown high lead exposure in children. The mean BLLs reported was 80 μg/L (geometric) in Yaoundé, Cameroon [35], 70 μg/L in urban South Africa [36], and 70 μg/L in Kampala, Uganda, and 80 μg/L in urban Kinshasa, Democratic Republic of Congo (DRC) [16]. In terms of the prevalence of elevated BLLs, recent studies have shown that 11% to 88% of children presented with BLLs beyond 50 μg/L. The proportion of children with BLLs beyond 50 μg/L was 11.4% in Nigeria [37], 74% in urban South Africa [36], and 88% in Cameroon [35].

While the proportions of children with BLLs beyond 100 μg/L reported in three studies carried out in SSA were 20.5% in Kampala, Uganda [24], 32% in Yaoundé, Cameroon [35], and 41% in urban Kinshasa, DRC [16]. These proportions are greater than the proportion (8.2%) found in our population at age 6 years.

### 4.1. BLLs and Sources of Lead at Age 1 and 6 Years 

Potential sources of lead including consumption of bushmeat killed by lead bullets positively associated with child BLLs at 1 year of age were still present at 6 years of age, with no significant difference in prevalence. Maternal use of eye cosmetics increased from 16.4% at age 1 year to 44.4% at age 6 years. This difference is probably related to the addition of the definition of eye cosmetics in the questionnaire at 6 years of age. This might have led to differences in understanding and eventually reporting of Khol makeup by mothers. Elevated BLLs at age 6 could be explained by the presence of new or old sources of exposure. The reduction in the prevalence of elevated BLLs > 100 µg/L at 6 years of age may be partly explained by a reduction in hand-to-mouth behaviour at 6 years of age. Although the sources of lead, like paint, may still be present, changes in child behaviour (e.g., hand-to-mouth behaviour at older ages) probably decreased the exposure from leaded paint.

Persistently high BLLs among children at age 6 years could be explained by the persistence of sources of exposure. Indeed, under stable exposure conditions, BLLs may remain stable [38]. It is suggestive that children are exposed to multiple and continuous sources of exposure at an older age where increased mobility and activities of children (e.g., participation in outdoor occupation activities of parents) add to the risk of high exposure. Besides, these children have concurrent risk factors for high Pb absorption. Nutritional deficiencies are an important concern in these young children [39]. Increased lead exposure is concurrent with nutritional deficiencies, especially iron deficiency [38].

### 4.2. Association between Sociodemographic Characteristics and Sources of Lead

Populations with certain sociodemographic and socioeconomic characteristics are more susceptible to lead exposure [23]. For example, populations in countries with poor economic development are at the highest risk of household exposures [40], as cited in Kordas et al. [23]. Several studies in other parts of the world reported associations between sociodemographic characteristics and sources of lead, particularly in developed countries, where BLLs are associated with lower socioeconomic status [41]. In the USA, for example, children with low socioeconomic status have been found to have higher blood lead levels as compared to children with higher socioeconomic status [41]. In this current study, sources of lead were not associated with the child’s sex, family’s wealth score, and maternal education level. However, families with higher socioeconomic status versus lower socioeconomic status reported more frequent presence of paint. In terms of populations at risk, interventions to decrease lead exposure should target all of the children, as there is no known toxicity threshold [42,43,44,45].

### 4.3. Dietary Sources of Lead

#### 4.3.1. Bushmeat Harvested with Lead Ammunition

Ammunition-derived lead is a significant source of dietary lead exposure in populations who eat wild game meat or bushmeat [13]. The populations at risk are mostly hunters, shoot employees, and their families, and their children [46]. This was also identified as a possible dietary source of lead in this population at age 1 year [26]. Other studies mainly carried out in developed countries outside SSA have shown that the concentration of lead in meals prepared from the wild-shot or hunted game had high levels of lead [47,48]. Studies have also shown positive associations between consumption of game meat killed by lead ammunition and BLLs in humans [28,49] or the presence of high risk of lead exposure from consumption of game meat [27,49,50].

Bushmeat is widely eaten across West Africa, including Benin. We previously described how it is hunted, processed, sold in the markets and consumed within families [51]. Bushmeat was banned in West Africa following the epidemic of Ebola Virus Disease (EVD) in 2013–2016 [29]. The primary purpose of the ban was to control and reduce the transmission of EVD through bushmeat. Possibly dietary practices and local attitudes toward bushmeat consumption changed in response to the EVD epidemic that emerged in March 2014 in West Africa. Indeed, few studies in other western African countries [52,53] reported a reduction of consumption of bushmeat during the EVD crisis. The prevalence of bushmeat consumption, in general, did not change after the EVD crisis in our study population (41.2% in 2011–2013 vs. 42.8% in 2016–2018). However, only 18.8% of families reported current bushmeat consumption in 2016–2018. The quantity and frequency of meat consumed may have decreased between the two periods of study (2011–2013 and 2016–2018) because of bushmeat ban.

#### 4.3.2. Lead in other Food Types

Consumption of peanuts and rice were significantly associated with increased 25th and 50th percentiles of BLLs, respectively, although the significance was marginal in the sensitivity analyses for rice. Lead could be transferred from soil to plant especially if the soil on which food is grown is from a former industrial site, or is next to old buildings or busy roads [54]. It could also be introduced in other ways during growth/production, transportation, preparation, and storage [54]. Previous studies have found high concentrations of lead in vegetables and concluded that they can pose a potential health risk to their consumers [55,56,57]. Recent studies from SSA have reported lead contents in rice and cereal products. The first multi-centre regional SSA Total Diet Study (TDS) (Benin, Mali, Cameroon, and Nigeria) showed that staple food (food routinely consumed) including meat, cereals and tubers (for example, peanuts, and rice) were contaminated with lead [58]. The concentrations of lead in Nigerian rice grains exceeded some Nigerian and international standards [59]. Tirima et al. [60] reported dietary contamination as a pathway for lead exposure in children during the 2010–2013 lead poisoning epidemic in Zamfara, Nigeria. This study assessed dietary lead exposure due to mainly contamination of staple cereal grains and legumes associated with a local artisanal gold mining.

Consumption of sweet potatoes was associated with decreased BLLs in sensitivity analyses. Sweet potatoes are often eaten after school by children, as well as yams, depending on the season. It is possible that this negative association only reflects the absence of consumption of yam. Indeed, according to the TDS mentioned above, yam may include high concentrations of lead.

### 4.4. Non-Dietary Sources

#### Activities with Lead

Activities involving potential use of lead and high or moderate risk of parental occupational exposure were associated with an increment in child BLLs with marginal significance. They were associated with an increase of the 90th percentile of BLLs, of 22.6 µg/L and 29.9 µg/L, respectively. This finding is consistent with other studies from Africa. A study conducted in automotive workshops in Ethiopia, reported mean BLLs (μg/L  ±  SD) of the automotive-garage workers to be 197.5 ± 4.46, which was significantly greater compared to a control group [61]. In another study carried out in South Africa, shooters had significantly elevated BLLs compared to archers (42.4% of shooters versus 5.9% of archers presented with BLLs ≥10 μg/dL) [62].

### 4.5. Strengths and Limitation

To our knowledge, this study is the only prospective children cohort in Benin, which monitored BLLs and sources of lead in young children. Very few studies in SSA and, to our knowledge, none in Benin, followed up BLLs in children. This enables the comparison of sources at different ages. Every attempt was made to reduce biases by assessing the same sources of lead with the same questions at 1 and 6 years of age. This study used data from a relatively large sample of children as compared to most previous studies in SSA. Finally, application of quantile regression was very useful in identifying specific sources that contributed to lower and higher levels of lead. The drawback is that quantile analysis decreases statistical power due to lower sample size; therefore, the interpretation shall not be limited to significant associations when the population size is limited as in our case. This study did not intend to assess the prevalence of elevated BLLs in a representative sample of Beninese children. Because children lost-to-follow-up presented higher BLLs at age 1 year compared with children followed at 6 years of age, the prevalence of BLLs at age 6 years could have been underestimated. The absence or observing a marginal significance between some potential sources of lead and BLLs could be due to a lack of statistical power (presence of paint in the house, use of eye cosmetics, activities involving potential use of lead, occupational risk) especially in the quantile regressions. In terms of sources, information on the consumption of all food items could not be assessed. There are other potential sources, for example, house dust, which could not be evaluated at age 6. However, house dust was possibly a source of Pb in this cohort at age 1 year (2011–2013) [26], as also reported in other studies [63,64].

In terms of future research needs, children should be further followed up to monitor BLLs at regular intervals. Moreover, the health outcomes associated with lead exposure should be studied in these children. Currently, there are limited epidemiological studies aimed at investigating the impact of lead exposure and child growth in SSA. In this regards, future research will investigate associations between childhood BLLs and different growth parameters in these children.

## 5. Conclusions

We confirmed persistently elevated blood lead levels in 6-year-old children residing in a semi-rural area in the south of Benin. Blood lead levels of children were strongly associated with several dietary sources. Main potential sources of lead identified included consumption of bushmeat harvested with lead ammunition and consumption of peanuts. These sources are frequent in this population and were not associated with sociodemographic characteristics. These results reinforce the importance of prevention programs, including further research and surveillance to reduce and eliminate lead exposure in children.

## Figures and Tables

**Table 1:** Study population characteristics of children aged 6 years, 2016–2018 in Benin (N = 425).

Variable	Category	*n*/*N*^a^	% or Mean ± SD
Health centre location			
	Attogon/Allada	150/425	35.3
	Sékou	275/425	64.7
**Family characteristics**			
Father’s education			
	None	157/417	37.7
	Some ^a^	260/417	62.4
Mothers education			
	None	264/419	63.0
	Some ^a^	155/419	37.0
Language spoken at home			
	Fon	186/421	44.2
	Aïzo	223/421	52.9
	Others	12/421	2.9
Socioeconomic status			
	Lowest	144/421	34.2
	Medium	151/421	35.9
	Highest	126/421	29.9
The child lives in collective housing			
	Yes	328/422	77.7
	No		
Parents smoking			
	Yes	17/407	4.2
	No	390/407	95.8
Child characteristics			
Sex			
	Boy	212/425	49.9
	Girl	213/425	50.1
Attending school			
	Yes	281/422	66.6
	No	141/422	33.4
Age at assessment (years)		425/425	6.2 ± 0.3

^a^ defined as completion of primary or higher level.

**Table 2:** Comparisons of sources of lead between age 1 and 6 years in the cohort of children in Benin.

Potential Sources	Age 1 Year (2011–2013)	Age 6 Years (2016–2018)	*P* ^a^
Bushmeat consumption ^b^	156/379 (41.2)	135/325 (41.5)	0.85
Presence of high or moderate risk of paternal occupational exposure	77/419 (18.4)	79/724 (18.6)	0.69
Use of pipe system for drinking water	308/379 (81.3)	371/423 (87.7)	<0.01
Presence of paint in the house	59/423 (14.0)	56/421 (13.3)	0.69
Presence of paint chips	19/379 (5.0)	23/421 (5.5)	0.86
Maternal use of eye cosmetics (Khol)	62/379 (16.4)	187/421 (44.4)	<0.01

All data are reported as *n* (%) ^a^ McNemar’s test ^b^ the same question at age 1 and 6 years was: “Does your child eat meat from animals killed by a rifle”.

**Table 3:** Multivariable analyses identifying potential sources of lead at different percentiles of BLLs in 6-year-old children in Benin, 2016–2018 (*n* = 390).

Potential Sources	*n* (%)	Expected % Difference in BLLs Compared with the Referent Group ^a^		Expected Difference in the Percentiles of BLLs µg/L Compared with the Referent Group							
				(95% CI) ^a^							
		% (95% CI)	*p*	25th Percentile	*p*	50th Percentile	*p*	75th Percentile	*p*	90th Percentile	*p*
Currently consuming meat killed by lead bullets (vs. no)	88 (18.8)	**13.3 (1.7, 26.2)**	0.02	1.6 (−4.1; 7.2)	0.6	3.8 (−2.6; 10.2)	0.2	**13.6 (0.4; 26.9)**	0.04	16.3 (−15.9; 48.5)	0.3
Consumption of rice (vs. less than 1–3 times/months or less)											
1–3 times/week	106 (25.1)	8.9 (−8.6.3, 29.7)	0.3	2.5 (−6.6; 11.6)	0.6	4.7 (−5.6; 15.0)	0.4	10.1 (−11.3; 31.4)	0.4	3.1 (−49.0; 55,3)	0.9
4–6 times/week	103 (24.4)	**19.6 (0.4, 42.5)**	0.05	2.6 (−6.5; 11.7)	0.6	10.2 (−0.2; 20.5)	0.06	**18.0 (−3.4; 39.4)**	0.1	9.3 (−42.9; 61.6)	0.7
At least once/day	179 (42.3)	**17.6 (-0.2, 38.7)**	0.05	3.9 (−4.7; 12.5)	0.4	**6.2 (−3.2; 16.2)**	**0.2**	13.8 (−6.3; 34.0)	0.2	1.4 (−47.8; 50.5)	1.0
Consumption of peanuts (referent category: never)											
<1/month	137 (32.8)	3.0 (−6.5, 13.4)	0.6	3.4 (−1.6; 8.4)	0.2	0.7 (−5.0; 6. 4)	0.8	1.7 (−10.1; 13.5)	0.8	3.1 (−25.7; 31.8)	0.8
>1/month	41 (9.8)	**22.0 (4.6, 42.5)**	0.01	**9.2 (1.1; 17.2)**	0.03	5.3 (−4.0; 14.0)	0.3	10.0 (−8.9; 28.9)	0.3	**73.0 (26.9; 119.1)**	<0.01
Consumption of sweet potatoes (referent category: never)											
<1/month	204 (48.2)	−7.7 (−16.6, 2.3)	0.8	1.6 (−3.7; 7.0)	0.6	−4.7 (−10.7; 1.3)	0.2	−8.0 (−20.5; 4.5)	0.2	−19.3 (−49.8; 11.3)	0.2
>1/month	97 (22.9)	−6.8 (−17.6, 5.4)	0.8	−0.9 (−7.3; 5.5)	0.8	−4.5 (−11.7; 2.7)	0.2	−9.2 (−24.3; 5.9)	0.2	−12.1 (−48.8; 24.7)	0.5
Presence of activity with potential use of lead (vs. absence)	61 (14.4)	**12.3 (−0.6, 26.8)**	0.06	4.4 (−1.9; 10.8)	0.2	3.0 (−4.2; 10.2)	0.4	6.4 (−8.5; 21.3)	0.4	22.6 (−13.8; 58.9)	0.2
Presence of high or moderate risk of paternal occupational exposure (vs. absence)	79 (18.6)	5.8 (−5.2, 18.1)	0.3	−1.2 (−6.9; 4.5)	0.7	**−4.2 (−10.7; 2.2)**	0.2	4.7 (−8.7; 18.1)	0.5	**29.9 (−2.9; 62.6)**	0.07
Presence of paint in the house (vs. absence)	56 (13.3)	4.7 (−7.8, 19.1)	0.5	4.6 (−2.0; 11.3)	0.2	−1.2(−8.7, 6.3)	0.8	2.7 (−13.0; 18.4)	0.7	12.5 (−25.7; 50.7)	0.5
Use of bore hole/cement/dug well for water source (vs. absence)	188 (44.4)	−4.4 (−3.2, 5.9)	0.5	−0.7 (−5.3; 4.0)	0.8	−1.1 (−6.4; 4.2)	0.7	−2.9 (−13.9; 8.0)	0.6	−6.2 (−32.9; 20.5)	0.7

^a^ adjusted for sex and family wealth quartiles. The cells with significant differences (*p* < 0.05) are in bold and highlighted in light grey. The cells with *p* < 0.2 are in bold only.

## Supplementary Materials

The following are available online at https://www.mdpi.com/1660-4601/17/22/8689/s1, Table S1: Comparison of characteristics at age 1 year between children to assess differences among included and excluded children at 1 year of age, Table S2: Bivariate analysis between sociodemographic factors and potential sources of lead in children at age 6 years (*N* = 425), Table S3: Associations between sources of lead and socio-demographic factors. Table S4: Sensitivity analysis—Multivariable analyses identifying potential sources of lead in 6-year-old children in Benin, 2016–2018, including all children assessed at age 6 years (*n* = 478).
