# Supplementary material for: Follow-Up of Elevated Blood Lead Levels and Sources in a Cohort of Children in Benin
Source: Int J Environ Res Public Health. 2020 Nov 23;17(22):8689. doi: 10.3390/ijerph17228689 (PMC7700695; doi:10.3390/ijerph17228689)
Supplement: Supplementary file 1 [file ijerph-17-08689-s001.pdf]

Table S1. Comparison of characteristics at 1 year between children to assess differences among included and excluded children at 1 year of age.

Characteristics	Children Assessed at Age 1 Year Only *N*= 260 *n* (%)	Children Assessed at Age 1 and 6 Years *N* = 425 *n* (%)	*P* [a]
BLLs>50 μg/L	163 (62. 7)	234 (55.1)	0.05
BLLs>100 μg/L	54 (20.8)	70 (16.5)	0.2
Bushmeat consumption	78 (34.8)	156 (41.2)	0.1
Child drinks water from the pipe system	191 (85.3)	308 (81.3)	0.2
Presence of paint in the house	37 (14.3)	59 (14.0)	0.9
Presence of paint chips in the house	16 (7.1)	19 (5.0)	0.3
Gasoline stored at home	156 (69.6)	297 (78.4)	0.02
Maternal use of eye cosmetics	51 (22.8)	62 (16.4)	0.05
Paternal high or moderate risk of occupational exposure	55 (22)	77 (18.4)	0.3
Activity involving potential use of lead	16 (7.1)	34 (9.0)	0.4
**Sociodemographic characteristics**			
Child's sex (male)	121 (46.9)	212 (50.1)	0.4
Wealth score quartiles			
lower	89 (35.0)	97 (22.9)	<0.01
median	53 (20.9)	150 (35.5)	
higher	50 (19.7)	71 (16.8)	
highest	62 (24.4)	105 (24.8)	
Child lives in collective housing	216 (85.0)	361 (85.3)	0.9
**Health centre location**			
Sekou	145 (56.2)	276 (65.3)	0.02
Attogon/Allada	113 (43.8)	147 (34.8)	

[a] Pearson chi-square.

Table S2. Bivariate analysis between sociodemographic factors and potential sources of lead in children at 6 years (*N* = 425).

Independent variable	*n*/*N* [a] (%)	% Change in BLLs compared with the referent group (95% CI)	Crude OR for 90th percentile of BLLs compared with the referent group (95% CI)
**Socio-demographic characteristics**			
Male sex (vs. female)	212/425 (49.9)	11.4 (2.6, 20.9) *	2.8 (1.4, 5.6) *
Some maternal education (vs. none) †	155/37.0	−4.3 (−12.2, 4.4)	0.8 (0.43, 1.64)
Quartiles of wealth score (vs. lower quintile)			
lower	144/421 (34.2)		
median	102/421 (24.2)	**7.8 (−17.4, 2.9)**	**0.5 (0.20, 1.20)**
high	116/421 (27.6)	1.1 (−9.0, 12.4)	0.8 (0.35, 1.64)
highest	59/421 (14.0)	**12.1 (−22.9, 0.2)**	0.5 (0.16, 1.47)
Potential sources of lead			
Bushmeat consumption (vs. no consumption)	135/325 (41.5)	5.8 (−4.2, 16.8)	1.5 (0.7, 3.0)
Currently consuming meat killed by lead bullets (vs. never)	88/399 (18.8)	**15.9 (4.2, 28.9)**	**1.8 (0.9, 3.8)**
Use of pipe system for water source (vs. absence)	371/423 (87.7)	2.5 (−9.6, 16.3)	1.4 (0.5, 4.2)
Use of bore hole/cement/dug well for water source (vs. absence)	188/423 (44.4)	**−6.2 (−13.7, 1.9)**	0.8 (0.44, 1.16)
Consumption of peanuts			
never	240/418 (57.4)		
<1 / month	137/418 (32.8)	1.5 (−7.3, 11.2)	0.6 (0.3, 1.3)
>1 / month	41/418 (9.8)	23.6 (7.1, 42.7) *	1.7 (0.7, 4.2)
Consumption of beans			
<1 / month +	61/423 (14.4)		
>1 / month	362/423 (85.6)	6.9 (−5.0, 20.2)	1.7 (0.6, 4.9)
Consumption of rice			
1–3 times/month or less	35/423 (8.3)		

1–3 times /week	106/423 (25.1)	6.0 (−10.2, 25.0)	0.6 (0.2, 2.7)
4–6 times/week	103/423 (24.4)	88.8 (0.7, 40.3) *	1.7 (0.5, 6.2)
At least once/day	179/423 (42.3)	**14.2 (−2.4, 33.6)**	1.3 (0.4, 4.5)
Consumption of sweet potatoes			
never	122/423 (28.8)		
<1 / month	204/423 (48.2)	**−9.0 (−17.4, 0.3)**	0.5 (0.2, 1.0) *
>1 / month	97/423 (22.9)	5.1 (−15.5, 6.5)	0.8 (0.4, 1.8)
Consumption of Cassava *(Manioc)*			
<1 / month ++	176/422 (41.7)		
>1 / month	246/422 (58.3)	−0.6 (−8.6, 8.1)	1.10 (0.6, 2.3)
Ever ingested soil or other products [b] (vs. no)	174/418 (40.9)	−15.1(−55.9, 63.6)	0.9 (0.4, 1.6)
Child has hand-to-mouth-behavior (vs. no)	135/421 (32.1)	3.4 (−5.3, 13.0)	1.1 (0.5, 2.1)
House in mud (vs. not in mud)	154/422 (36.5)	−0.8 (−9.0, 8.1)	1.1 (0.6, 2.1)
Presence of paint in the house	56/421 (13.3)	**6.8 (−5.5, 2.7)**	**1.9 (0.9, 4.3)**
Presence of paint chips in the house (vs. absence)	23/421 (5.5)	26.2 (4.0, 53.2) *	1.4 (0.4, 4.9)
Use of artisanal cookware (made from recycled material)	403/422 (95.5)	6.9 (−12. 5, 30.6)	2.0 (0.3, 15.7)
Use of artisanal cookware (made from terracotta/clay)	59/418 (14.1)	4.2 (−7.6, 17.5)	0.8 (0.3, 2.2)
Child use eye cosmetics *(Khol) (vs. no use)*	58/413 (14.04)	0.2 (−11.3, 13.2)	1.0 (0.4, 2.6)
Presence of activity with potential use of lead [c] (vs. absence)	61/423 (14.4)	**11.6 (0.0, 26.0)**	2.7 (1.3, 5.6) *
Presence of high or moderate risk of paternal occupational exposure (vs. absence)	79/424 (18.6)	6.5 (−4.2, 18.5)	2.8 (1.4, 5.5) *
Residence at 200m of heavy traffic	327/418 (78.2)	1.00 (0.5, 2.1)	1.00 (0.5, 2.1)

[a] Totals may not equal to 260 and 425 due to missing data [b] products include earth, kaolin, kalaba, termite mound [c] activities include metal smelter, battery recycling/storage, radiator repair, metal recycling/storage, lead solder, vehicle repair, manufacturing of ammunition/metal objects * $p < 0.05$, Beta coefficients in bold only have $p < 0.2$ + includes 9 never consumers, ++ includes 21 never consumers. † defined as completion of primary or higher level.

Table S3. Associations between sources of lead and socio-demographic factors.

	**Consumption of bushmeat** ***n*** **(%)**	***P***	**Rice more than 4–6 times per week (vs. less)** ***n*** **(%)**	***P***	**Peanut more than once per month (vs. less)** ***n*** **(%)**	***P***	**Presence of paint** ***n*** **(%)**	***P***	**Presence of occupational exposure** ***n*** **(%)**	***P***	**Presence of activities with lead** ***n*** **(%)**	***P***
**Sex**												
Boys	38 (19.1)	0.6	142 (67.3)	0.8	20 (9.7)	0.9	29 (12.8)	0.8	42 (19.8)	0.5	31 (14.7)	0.9
Girls	42 (21.1)		140 (66.0)		21 (10.0)		27 (13.8)		37 (17.5)		30 (14.2)	
**Family wealth quartiles**												
lower	29 (21.5)	0.7	97 (67.4)	0.4	15 (10.6)	0.1	13 (9.2)	0.01	23 (16.1)	0.6	24 (16.7)	0.4
median	20 (21.1)		65 (63.7)		5 (4.9)		8 (7.8)		20 (19.6)		16 (15.7)	
higher	22 (20.0)		84 (72.4)		17 (14.9)		21 (18.1)		21 (18.1)		11 (9.5)	
highest	8 (13.8)		36 (61.0)		4 (6.9)		14 (23.7)		14 (23.7)		10 (17.0)	
**Maternal education**												
None	51 (20.7)	0.5	176 (66.7)	0.9	22 (8.33)	0.2	27 (10.3)	0.02	45 (17.1)	0.4	43 (16.3)	0.2
Some	26 (17.7)		103 (66.6)		19 (12.7)		29 (18.7)		32 (20.7)		18 (11.6)	

* Pearson chi-square test.

Table S4. Sensitivity analysis—Multivariable analyses identifying potential sources of lead in 6-year-old children in Benin, 2016–2018, including all children assessed at 6 years ($n = 478$).

**Potential sources**	***n*** **(%)**	**Expected % Difference in BLLs Compared with Referent Group** [a]		**Expected Difference in the Percentiles of BLLs μg/L Compared with the Referent Group (95% CI)** [a]							
		**% (95% CI)**	***p***	**25th percentile**	***p***	**50th percentile**	***p***	**75th percentile**	***p***	**90th percentile**	***p***
Currently consuming meat killed by lead bullets (vs. no)	85 (19.5)	**10.2 (2.0, 19.1)**	0.01	1.9 (−3.6; 7.4)	0.5	3.1 (−1.6; 7.2)	0.2	12.2 (−0.4; 18.7)	0.06	11.4 (−14.7; 37.5)	0.4
Consumption of rice (referent category: less than 1-3 times/months or less)											
1-3 times/wk	119 (25.3)	9.0 (−7.3, 28.2)	0.3	3.0 (−5.8 ; 11.8)	0.5	1.9 (−7.4; 11.2)	0.7	11.5 (−8.6; 31.7)	0.3	4.0 (−38.0; 46,0)	0.9
4-6 times/wk	118 (25.1)	**17.1 (−0.5, 37.9)**	0.06	1.8 (−7.1;10.7)	0.7	7.0 (−2.4; 16.4)	0.1	19.0 (−1.3; 39.4)	0.07	9.9 (−32.4; 52.2)	0.7
at least once/day	196 (41.7)	**15.1 (−1.4, 34.4)**	0.08	3.9 (−4.6; 12.3)	0.4	1.9 (−7.0; 10.8)	0.7	14.1 (−5.1; 33.4)	0.2	5.5 (−34.6; 45.6)	0.8
Consumption of peanuts (referent category: never)											
<1 / month	152 (32.8)	2.4 (−6.1, 11.7)	0.6	2.4 (−2.3; 7.2)	0.3	−0.5 (−5.5; 4.5)	1.0	1.5 (−9.4; 12.3)	0.8	4.1 (−18.4; 26.6)	0.7
>1 / month	43 (9.3)	**20.1 (4.3, 38.4)**	0.01	**6.6 (−1.1; 14.3)**	0.09	5.7 (−2. 4; 13.8)	0.2	11.1 (−6.5; 28.7)	0.2	**46.8 (10.2; 83.4)**	0.01
Consumption of sweet potatoes (referent category: never)											
<1 / month	226 (48.1)	−7.7 (−15.8, 1.3)	0.1	0.7 (−4.5; 6.0)	0.8	**−5. 9 (−11.2; −0.6)**	0.03	−6.1 (−17.6; 5.4)	0.3	−14.4 (−38.3; 9.5)	0.2
>1 / month	107 (22.8)	−7.7 (17.4, 3.1)	0.2	0.1 (−6.2; 6.4)	1.0	−6.2 (−12.5; 0.1)	0.06	−6.8 (−20.6; 7.0)	0.3	−13.0 (−41.7; 15.7)	0.4

Presence of activity with potential use of lead (vs. absence)	67 (14.3)	**13.3 (1.3, 26.6)**	0.03	3.9 (−2.2; 10.0)	0.2	**6.8 (0.4; 13.2)**	0.04	7.4 (−6.4; 21.3)	0.3	−19.4 (−9.5; 48.3)	0.2
Presence of high or moderate risk of paternal occupational exposure (vs. absence)	81 (18.4)	6.7 (−3.4, 17.9)	0.2	−0.8 (−6.3; 4.6)	0.8	−3.3 (−9.0; 2.4)	0.3	6.2 (−6.2; 18.7)	0.3	10.2 (−15.6; 36.0)	0.4
Presence of paint in house (vs. absence)	63 (13.5)	3.6 (−2.7, 9.9)	0.3	3.6 (−2.7; 9.9)	0.3	0.9 (−5.8; 7.6)	0.8	3.4 (−11.1; 17.9)	0.6	16.3 (−13.8; 46.4)	0.3
Use of bore hole/cement/dug well for water source (vs. absence)	210 (44.7)	−1.4 (−5.8, 2.9)	0.5	−1.1 (−6.0; 3.8)	0.5	−1.3 (−5.9; 3.3)	0.6	−3.1 (−13.2; 6.9)	0.5	−2.2 (−23.1; 18.7)	0.8

[a] adjusted for sex and family wealth quartiles. The cells with significant differences ($p < 0.05$) are highlighted in light grey. The cells with $p < 0.2$ are in bold only.
